# Addition of silver nanoparticles to the zinc ferrite/polyaniline composition for boosting its visible photocatalytic degradation

**DOI:** 10.1039/d4ra05096g

**Published:** 2024-08-19

**Authors:** Safanah Sahib Jaafar, Rana Ismael Faeq, Amel Muhson Naji, Olfat A. Nief, Mustafa K. A. Mohammed

**Affiliations:** a Department of Chemistry, College of Science, Mustansiriyah University P. O. BOX 14132 Baghdad Iraq; b Department of Optics Techniques, Dijlah University College Al-Masafi Street Baghdad 00964 Iraq; c College of Remote Sensing and Geophysics, Al-Karkh University of Science Baghdad 10011 Iraq mustafa_kareem97@yahoo.com

## Abstract

Enhancing the photocatalytic activity of ZnFe_2_O_4_ with a good energy band gap to degrade industrial waste under sunlight illumination can help to develop green environments. Here, to improve the photocatalytic efficiency of ZnFe_2_O_4_ ferrites, they were merged with polyaniline (PAni) and silver (Ag) nanoparticles to synthesize Ag@ZnFe_2_O_4_–PAni plasmonic nanostructures. The as-synthesized nanostructures were characterized using a series of advanced characterization techniques to confirm successful formation and investigate photocatalytic improvement origins. It was found that incorporating Ag NPs along with the PAni to ZnFe_2_O_4_ increases its absorption power and red-shifts its energy band gap, which increases the electron–hole production rate by exposure to light in ZnFe_2_O_4_. Contribution of the surface plasmon resonance effect of Ag NPs and conjugated double bonds of PAni to charge transfer mechanisms in Ag@ZnFe_2_O_4_–PAni material increased charge separation during photocatalytic process, boosting the photodegradation performance of ZnFe_2_O_4_.

## Abstract 2

Enhancing the photocatalytic activity of ZnFe_2_O_4_ with a good energy band gap to degrade industrial waste under sunlight illumination can help to develop green environments.

## Graphical abstract

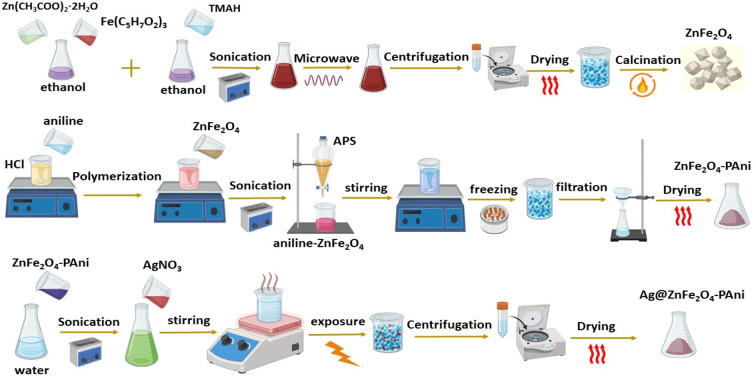

## 1 Introduction

With the increase in world population and the industrial development to meet human needs for food, clothing, electrical appliances, *etc.*, the increase in environmental pollution has become a critical challenge.^1,2^ This industrial waste affects the water quality of rivers and underground water, and subsequently negatively impact human health and the ecosystem.^3,4^ Heavy metal ions, phenols, dyes, polychlorinated biphenyls (PCBs), pharmaceutical drugs, haloacetic acids (HAAs), pesticides, disinfection byproducts (DBPs), and other synthetic chemicals are the main sources of water pollution.^5–8^ For years, the Environmental Protection Agency (EPA) and World Health Organization (WHO) have recommended that the world's industries do not discharge contaminated water into their surrounding environments. Various methodologies have been suggested to clean the water before discharging, including, precipitation, ozonation, adsorption, membrane separation, biodegradation, sonodegradation, solvent extraction, and ion exchange.^9–13^ These approaches have serious drawbacks, including toxic products, high operating costs, and low elimination rates. In contrast, advanced oxidation photocatalysis offers an ultimate deterioration, cheap, and eco-friendly method to treat wastewater with considerable recyclability.^14,15^ The photocatalysis process is used to disinfect the wastewater and eliminate contaminants from water and air. The photocatalyst material promotes the elimination reaction rate of targeted waste from water with the assistance of a light illumination source. During the process, the irradiated light reacts with the photocatalyst material to generate electron–hole pairs. Next, these photo-generated electrons participate in reduction reactions to start the oxidation of holes.^16–18^

In recent years, nano-sized materials were introduced as a primary area of research to find advanced technologies for meeting the world's demands. Nano-sized materials have a considerable surface area, which increases their chemical reaction with other materials. In addition, tunable electrical conductivity and optical properties of nano-sized materials along with their mechanical strength, compared to their bulk counterparts, makes them interesting materials to design different advanced technologies. Nanotechnology suggests cost-effective and efficient materials to use in the remediation of dirty water.^19–23^ As mentioned, industrial development and its conflict of interest with the green environment have led to an excessive increase in the pollution of natural water. Developing advanced nanomaterial photocatalysts has emerged as a promising technology to address this concern.^24^ Spinel ferrites have attracted environmental research due to their unique features, such as chemical stability, high adsorption capacities, cost-effective preparations, and superparamagnetic capabilities, to employ them as photocatalyst material for water treatment to remove pollutants *via* photodegradation mechanism.^25–29^ Zinc ferrite (ZnFe_2_O_4_), due to their small band gap, is more favorable for visible photocatalytic degradation of water pollution. However, the net zinc ferrites do not have strong photocatalytic efficiency due to weak charge separation.^30–32^

Yu *et al.*^31^ to enhance catalytic activity of zinc ferrites substituted copper metal to ZnFe_2_O_4_ structure. They deduced that the increased catalytic activity of zinc-based ferrite ascribes to the dual active sites of Fe and Cu and oxygen vacancies after Cu substitution. Janani *et al.*^32^ decorated ZnFe_2_O_4_ with CdO material to boost the photocatalytic efficiency of ZnFe_2_O_4_. They found that the CdO/ZnFe_2_O_4_ with a high surface area offers more active sites to induce the photocatalysis performance of the system. In addition, they concluded that coupling of ZnFe_2_O_4_ and CdO improved the lifetime of charge carriers and promoted the reaction of photo-generated electrons and holes with dye molecules. Akshhayya *et al.*^33^ decorated ZnFe_2_O_4_ with SnS_2_ material to accelerate visible light photocatalysis of methylene blue. They observed that the formed interfacial contact in the ZnFe_2_O_4_/SnS_2_ system suppresses charge recombination and retains better charge separation. These improvements with high visible-light absorbing ability offer an n-SnS_2_/p-ZnFe_2_O_4_ hybrid system with improved photocatalytic activity. Kaushal *et al.*^34^ developed a ZnFe_2_O_4_@nitrogen-doped carbon dots hybrid material to decomposite ciprofloxacin and norfloxacin materials under visible light irradiance. They observed that nitrogen-doped carbon dots hybridized with zinc ferrites, which enhanced photocurrent density and surface area. The ZnFe_2_O_4_@nitrogen-doped carbon dots exhibited efficient transfer of charge carriers and can be used for environmental remediation through the photocatalytic degradation process. Modification of ZnFe_2_O_4_ with conjugated polymers such as polyaniline (PAni) with considerable carriers mobility and good optical properties can induce its photocatalytic activity.^35–37^ Photosensitizer behavior of PAni under visible light illumination establishes the electron donor phenomenon in it, resulting in enhanced catalytic performance in PAni-contained hybrid heterostructures.^38,39^ Developing plasmonic nanostructures based on ZnFe_2_O_4_ also can be used to increase the ZnFe_2_O_4_ catalyst activity. The surface plasmon resonance (SPR) effect observed in noble metals usually increases the light-harvesting ability of heterojunctions, resulting in considerable enhancement in the catalytic and photocatalytic activity.^40–42^

The objective of the current study is to develop a ternary plasmonic nanostructures system based on ZnFe_2_O_4_ through its combination with PAni and Ag NPs. The Ag@ZnFe_2_O_4_–PAni hybrid system was employed for the visible light photocatalytic application and recorded promising results. Results showed that the Ag@ZnFe_2_O_4_–PAni nanocomposites have efficient charge transfer, higher surface area, and higher light-harvesting ability due to synergistic effects of Ag NPs and PAni, resulting in higher photodegradation behavior than the ZnFe_2_O_4_ and ZnFe_2_O_4_–PAni materials. Moreover, the effect of the pH value of aqueous mediums, the initial concentration of dye, and the amount of photocatalyst were examined on the photocatalytic performance of the Ag@ZnFe_2_O_4_–PAni.

## 2 Experimental

### 2.1 Materials

All solvents and materials that used in this study were purchased from Merck and used as received without any more purification steps.

### 2.2 Synthesis of zinc ferrite material

To synthesize ZnFe_2_O_4_, 1.8 mmol of Zn(CH_3_COO)_2_·2H_2_O and 2 mmol of Fe(C_5_H_7_O_2_)_3_ were dissolved in 10 mL of ethanol. In another vial, 10 mL of 2 mmol tetramethylammonium hydroxide pentahydrate (TMAH) solution in ethanol was prepared. Then, two solutions were added together and sonicated to obtain a dark red color solution. The obtained solution was exposed to microwave radiation at 180 °C for 45 min. The product was centrifuged and washed with ethanol. Finally, the brown precipitates were dried in a vacuum oven at 60 °C overnight, followed by calcination at 400 °C for 2 h to produce ZnFe_2_O_4_ NPs.

### 2.3 Synthesis of zinc ferrite–polyaniline material

To synthesize ZnFe_2_O_4_–PAni nanocomposite, an *in situ* polymerization technique was employed. 4 g of aniline material was dissolved into 200 mL of 1 M HCl solution by stirring for 2 h at a temperature of 50 °C. Then, 1.2 g ZnFe_2_O_4_ NPs were added to the aniline solution, followed by sonication for about 30 min at room temperature to homogenize the scattered ZnFe_2_O_4_ NPs. In another vial, 100 mL of 1 M HCl was carefully mixed with ammonium persulfate (APS) to obtain a 0.2 M solution. Then, the precooled APS solution was dropped wise to the aniline–ZnFe_2_O_4_ solution, followed by stirring at a temperature of 2 °C for 12 h to complete polymerization. The obtained solution was kept in the freezer overnight. Finally, the solution was filtered and washed with deionized water and methanol, followed by drying in a vacuum oven for 24 h at a temperature of 60 °C.

### 2.4 Synthesis of zinc ferrite–polyaniline decorated with silver nanoparticles

**Fig. 1:**
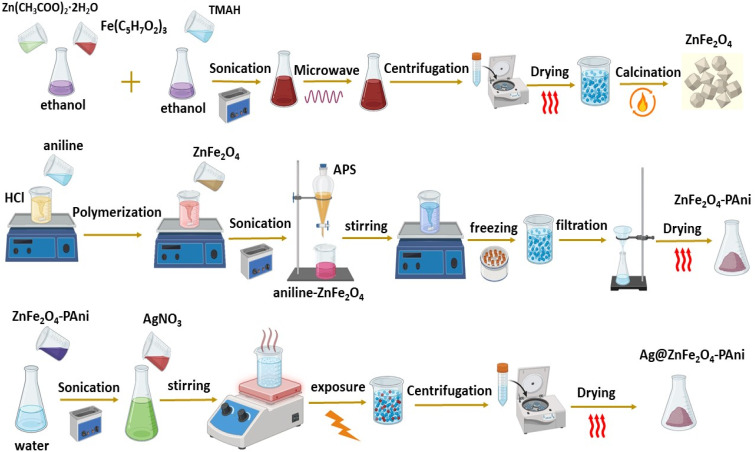
Schematic diagram for preparation of Ag@ZnFe_2_O_4_–PAni nanocomposite.

The Ag@ZnFe_2_O_4_–PAni plasmonic nanocomposites were synthesized through Ag^+^ ions photoreduction in the ZnFe_2_O_4_–PAni aqueous solution (see Fig. 1). 400 mg of ZnFe_2_O_4_–PAni nanostructures were sonicated in 100 mL deionized water for 30 min to prepare a homogeneous suspension. Then, 40 mL of 2.5, 5, and 10 mM of AgNO_3_ solution in deionized water were quickly added to ZnFe_2_O_4_–PAni aqueous solution in dark conditions. The suspensions were stirred for 2 h at room temperature in dark conditions and then exposed to natural sunlight for 90 min. The black precipitates were centrifuged and washed with deionized water and methanol repeatedly to remove impurities. The washed products were dried overnight in an oven at 60 °C for 24 h. The obtained powders were marked as AZP1, AZP2, and AZP3, referring to samples prepared using 2.5, 5, and 10 mM AgNO_3_ solutions, respectively.

### 2.5 Photocatalytic degradation investigation

The photocatalytic performance of the synthesized nanostructures for methylene blue (MB) or rhodamine B (RhB) dyes photodegradation was investigated by recording their UV-Vis spectra over reaction time under sunlight irradiance. For this test, 25 mg of MB or RhB dyes were dissolved into distilled water to obtain 25 ppm solutions. Then, 10 mg of photocatalyst materials (ZnFe_2_O_4_, ZnFe_2_O_4_–PAni, AZP1, AZP2, and AZP3) were dispersed into 100 mL dye solutions. The dye/photocatalyst material solutions were stirred under dark conditions for 30 min to establish an adsorption/desorption equilibrium between dye molecules and photocatalyst materials. Afterward, solutions were exposed to simulated sunlight illumination, and recorded their absorbance spectra. The same procedures were conducted to study the effects of pH and catalyst material concentration on the photodegradation performance of AZP2 material.

### 2.6 Characterizations

Crystalline structure of ZnFe_2_O_4_, ZnFe_2_O_4_–PAni, AZP1, AZP2, and AZP3 materials were measured by recording X-ray Diffraction (XRD) using a Rigaku Ultima IV XRD instrument. The morphology of the synthesized nanostructures were monitored using CM120 TEM and TESCAN Mira III FESEM. The FTIR spectra of materials were investigated using NICOLET-IS FTIR instrument. UV-vis spectra of samples were recorded using PerkinElmer Lambda Spectrometer. RAMAN spectra of materials were collected using a WiTec alpha 300 Raman spectrometer. The EIS response of samples were measured using Gamry Interface 1000 Potentiostat. PL spectra of material was collected using FLS 1000, Edinburgh spectrometer. Specific surface area of samples were measured by recording their Bet response using a BELSORP Mini II device. The transient photocurrent response of samples were measured using a PGSTAT302 N electrochemical workstation with a 500 W xenon lamp as the light source.

## 3 Results and discussion

**Fig. 2:**
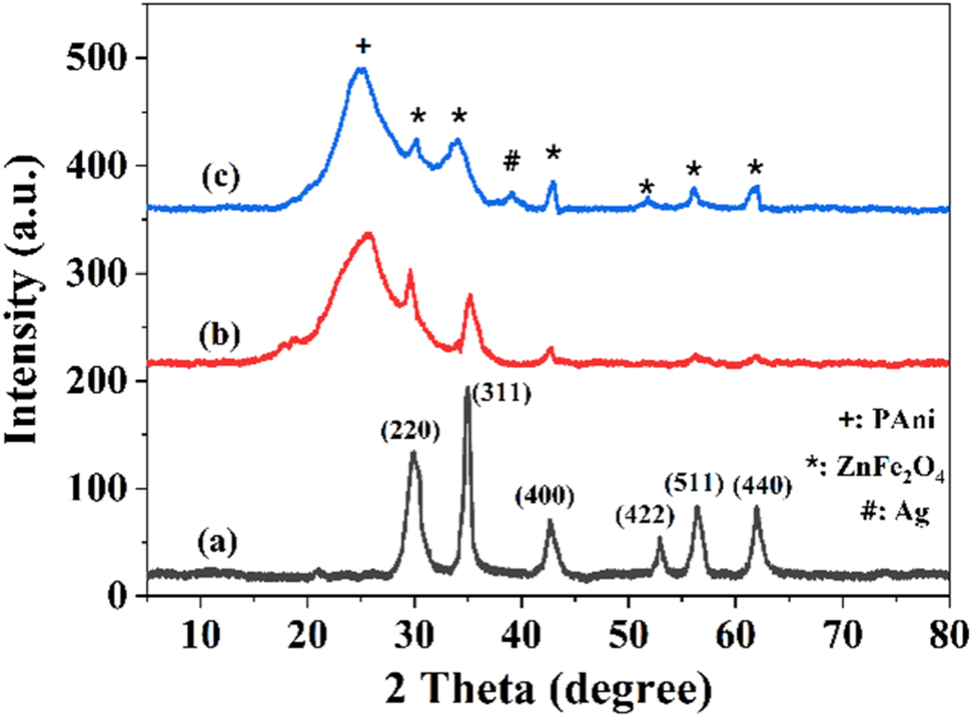
XRD spectra of (a) ZnFe_2_O_4_, (b) ZnFe_2_O_4_–PAni, and (c) AZP2 nanostructures.

Fig. 2 shows XRD patterns of different samples to confirm their successful synthesis. As shown in Fig. 2a, there are six peaks positioned at 29.90°, 35°, 42.6°, 52.9°, 56.4°, and 62°, which are assigned to the (220), (311), (400), (422), (511), and (440) planes, respectively. These peaks align with the JCPDS file No. 00-022-1, confirming the formation of ZnFe_2_O_4_ NPs.^43^ In the XRD pattern of ZnFe_2_O_4_–PAni (Fig. 2b), a broad peak at 25.7° is observed, attributing to the amorphous nature of PAni with the crystalline plane of (200). Besides, six peaks, referring to (220), (311), (400), (422), (511), and (440) planes of ZnFe_2_O_4_, are observed in the XRD pattern and prove the formation of ZnFe_2_O_4_–PAni hybrid structure. Fig. 2c shows XRD pattern of AZP2 material. In addition to the above-mentioned ZnFe_2_O_4_ and PAni phases, another peak is observed at 39.2° assigned to (111) Ag plane. It should be noted that the XRD peak of all the ZnFe_2_O_4_ NPs have minor shifts in AZP2, due to possible reaction of PAni and Ag with ZnFe_2_O_4_ material. These findings corroborate ternary Ag@ZnFe_2_O_4_–PAni plasmonic nanocomposites formation.

**Fig. 3:**
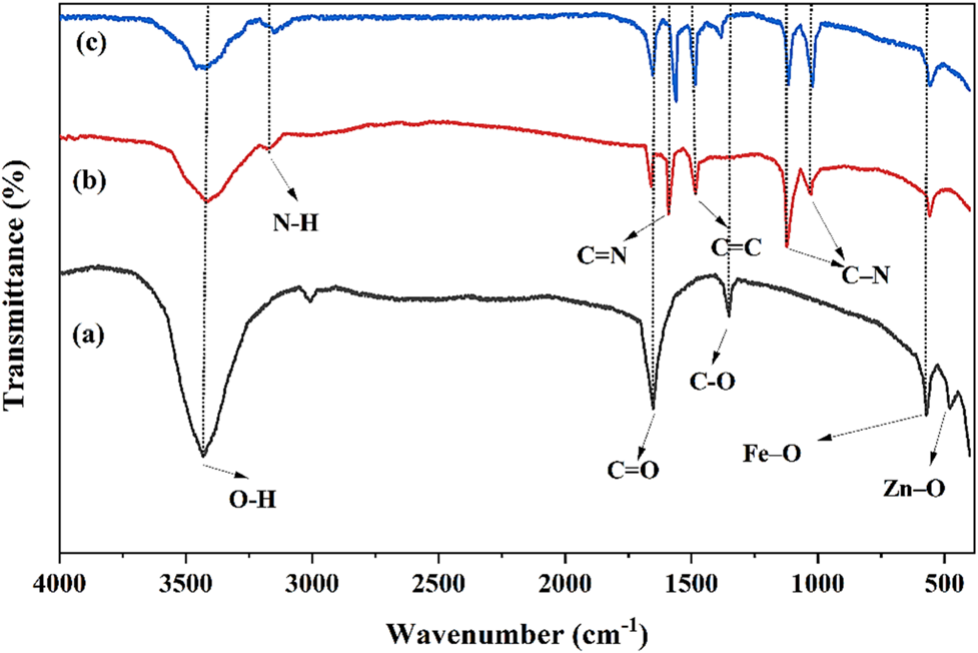
FTIR spectra of (a) ZnFe_2_O_4_, (b) ZnFe_2_O_4_–PAni, and (c) AZP2 nanostructures.

The FTIR spectra of ZnFe_2_O_4_, ZnFe_2_O_4_–PAni, AZP2 nanostructures are depicted in Fig. 3. The ZnFe_2_O_4_ spectrum encompasses five major peaks at 478.3 and 571.7 cm^−1^ attributed to vibrations of Fe–O and Zn–O bonds at octahedral and tetrahedral sites, 1351.5 cm^−1^ attributed to C–O stretching from residual synthesis precursors, and 1649.5 and 3430.9 cm^−1^ attributed to the bending and stretching vibrations of adsorbed water. In FTIR spectrum of ZnFe_2_O_4_–PAni (Fig. 2b), the characteristic peak of Fe–O in vanished, which can be caused by covering these bonds with the PAni molecules. In addition, five peaks at 1029.8 cm^−1^ assigned to secondary amine C–N stretching vibration, 1123.9 cm^−1^ assigned to vibration frequency of nitrogen quinone, 1484.2 cm^−1^ assigned to vibration for C=C bonds, 1593.1 cm^−1^ assigned to stretching mode of vibration for the C=N, and 3177.3 cm^−1^ assigned to the N–H stretching vibration. The AZP2 spectrum encompasses all peaks of ZnFe_2_O_4_–PAni with no more peaks. Notably, the nitrogen bonds shifted from 1129.7 cm^−1^ to 1021.8 cm^−1^ and N–H bonds shifted from 3177.3 cm^−1^ to 3146.7 cm^−1^, indicating successful incorporation of Ag NPs to ZnFe_2_O_4_–PAni nanostructures.

**Fig. 4:**
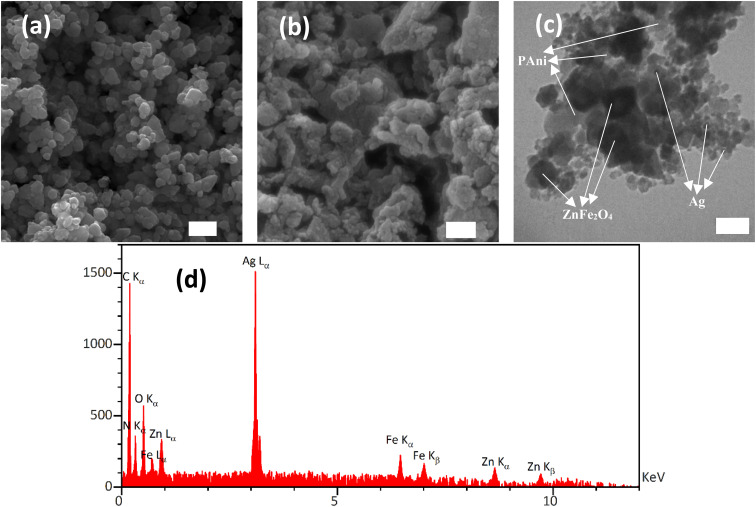
FESEM image of (a) ZnFe_2_O_4_ and (b) AZP2. TEM image of (c) AZP2. (d) EDS elements mapping images of AZP2 composite. Scale bar of FESEM and TEM images are 200 nm and 80 nm, respectively.

**Fig. 5:**
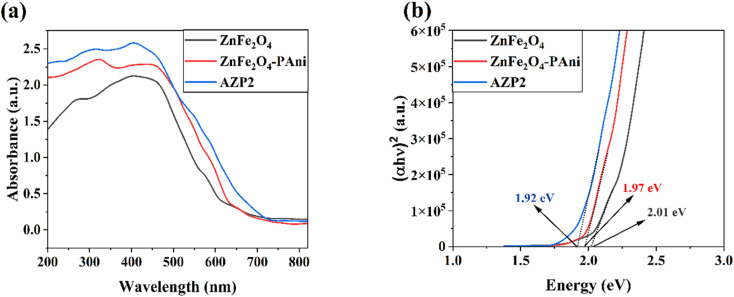
(a) Absorbance spectra and (b) corresponding Tauc plots of ZnFe_2_O_4_, ZnFe_2_O_4_–PAni, and AZP2 nanostructures.

The FESEM images of samples were taken to investigate the surface morphology of ZnFe_2_O_4_ and AZP2 photocatalysts (Fig. 4a and b). The nano-sized ZnFe_2_O_4_ particles were aggregated due to the magnetic attraction forces among ZnFe_2_O_4_ (Fig. 4a). In Fig. 4b, it is evident that particles are highly agglomerated, which is possibly due to the incorporation of Ag NPs. The successful incorporation of Ag NPs into the ZnFe_2_O_4_–PAni nanostructures was evaluated by TEM (Fig. 3c). As can be seen, ZnFe_2_O_4_ NPs were capped with PAni molecules. Small Ag NPs attached on the ZnFe_2_O_4_ or PAni surfaces. Fig. 4d shows the EDS spectrum of the Ag@ZnFe_2_O_4_–PAni nanostructure. As seen, six elements of C, N, O, Fe, Zn, and Ag were observed in the sample, consistent with existing elements in the Ag@ZnFe_2_O_4_–PAni composite. It proves successful synthesis of the Ag@ZnFe_2_O_4_–PAni. Fig. 5a shows UV-Vis spectra of as-prepared photocatalyst materials. As can be seen, by incorporating Ag NPs into ZnFe_2_O_4_–PAni nanostructure, its light-harvesting behavior is increased. This phenomenon increases the potential of ZnFe_2_O_4_ to absorb exposed light and generate electron–hole charges. Fig. 5b depicts the corresponding Tauc plot of samples to investigate their optical bandgap energy. The optical bandgap for ZnFe_2_O_4_ is 2.01 eV, aligning with the reported value in literature.^44^ Through the reaction of ZnFe_2_O_4_ with PAni materials, the optical bandgap is red-shifted to 1.97 eV and by the incorporation of Ag NPs to ZnFe_2_O_4_–PAni, it more reduced to 1.92 eV. It reveals that forming Ag@ZnFe_2_O_4_–PAni nanostructures can minimize the optical band gap of net ZnFe_2_O_4_ and increase light utilization, improving the photodegradation performance of ZnFe_2_O_4_ under visible light illumination.

**Fig. 6:**
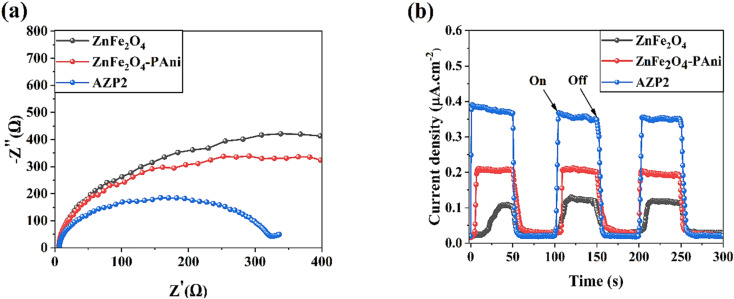
(a) EIS spectra and (b) transient photocurrent response of ZnFe_2_O_4_, ZnFe_2_O_4_–PAni, and AZP2 nanostructures.

Nyquist plots of samples using EIS method were obtained under light illumination to investigate the interfacial charge transfer resistance (Fig. 6a). By fitting the EIS spectra with the equivalent electrical circuit, parameters including series resistance (*R*_S_) and charge transport resistance (*R*_Ch_) were obtained. The EIS arc radius of AZP2 on the Nyquist plot is smaller than that of ZnFe_2_O_4_ and ZnFe_2_O_4_–PAni, which implies the AZP2 nanostructures have the lowest *R*_Ch_ value (87.8 Ω). It indicates a fast interfacial charge carrier transfer with high photogenerated carriers separation efficiency, which both are responsible for the enhanced photocatalytic activity of ZnFe_2_O_4_. It is due to the contribution of conjugated double bonds along the PAni structure and SPR effects of Ag NPs for the charge transfer mechanisms in AZP2 material. To get deeper insight on the photoelectrochemical properties of ZnFe_2_O_4_ NPs before and after modifications with PAni and Ag NPs, transient photocurrent response of ZnFe_2_O_4_ and ZnFe_2_O_4_–PAni, and AZP2 materials were investigated (Fig. 6b). As observed, the ZnFe_2_O_4_ sample implies a delayed photocurrent response upon the light is turned on and off. In contrast, the ZnFe_2_O_4_–PAni and AZP2 materials have rapid photocurrent responses. Moreover, results shows that the photocurrent of the ZnFe_2_O_4_–PAni electrode (0.213 mA cm^−2^) is 67.7% higher than that of ZnFe_2_O_4_ (0.126 mA cm^−2^), indicating the improved photoinduced electron–hole pairs separation efficiency in ZnFe_2_O_4_–PAni due to unique interfacial charge transfer of heterojunction. AZP2 shows the highest photocurrent density (0.358 mA cm^−2^), indicating that the charge carrier separation in modified nanostructure of Ag@ZnFe_2_O_4_–PAni is further improved.

**Fig. 7:**
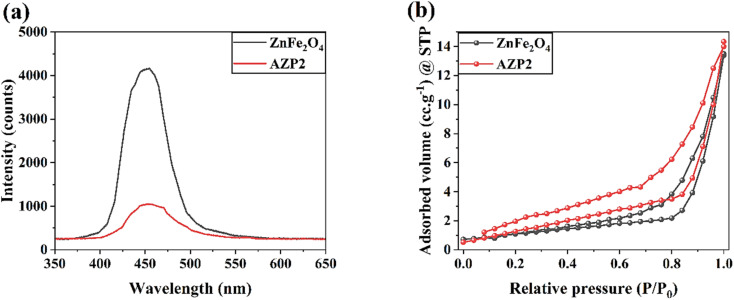
(a) PL spectra and (b) BET curves of ZnFe_2_O_4_ and AZP2 nanostructures.

**Fig. 8:**
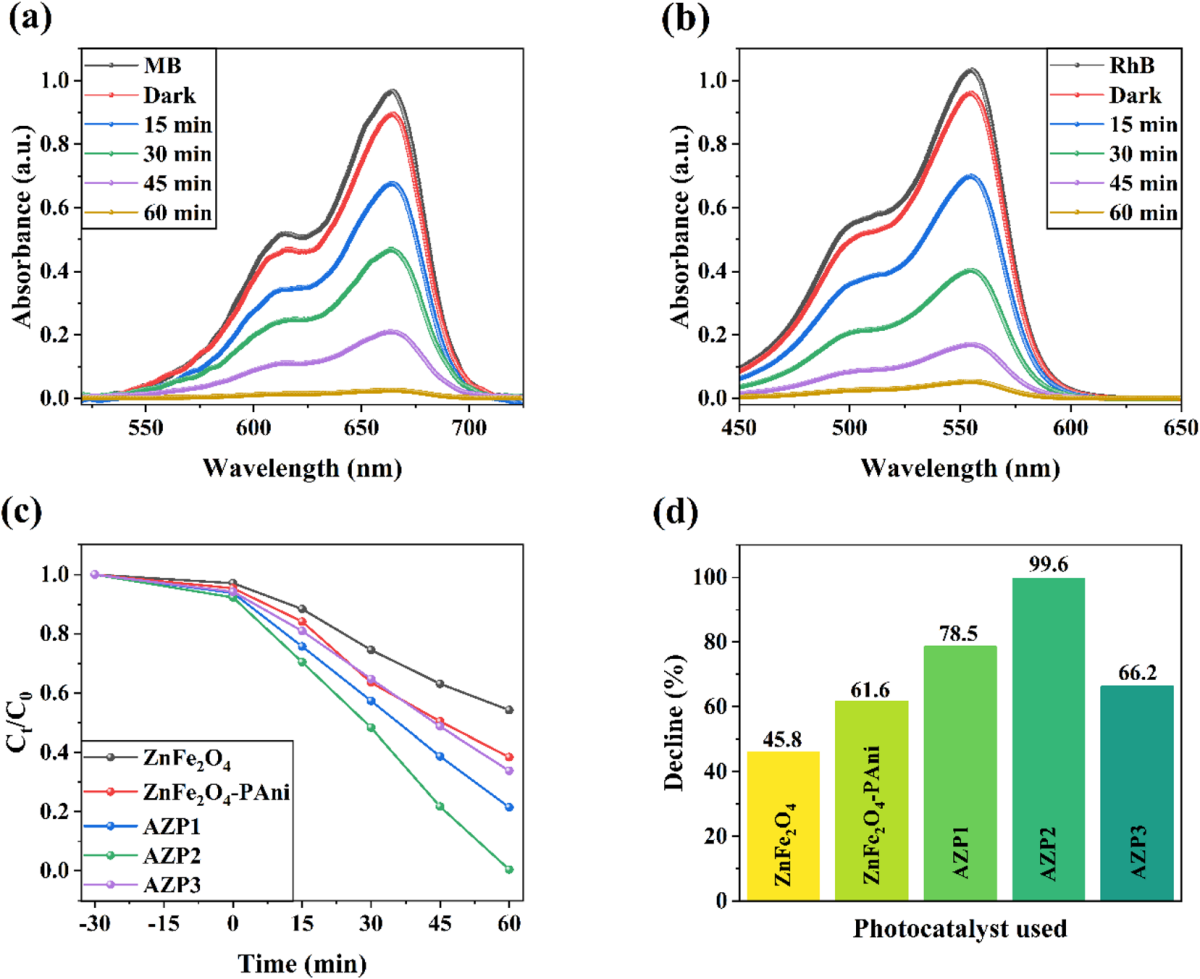
Photocatalytic activity of AZP2 for (a) MB and (b) RhB dyes. (c) Kinetic plot *versus* illumination time for MB of ZnFe_2_O_4_, ZnFe_2_O_4_–PAni, AZP1, AZP2, and AZP3 nanostructures. (d) Comparison of MB degradation over different photocatalyst.

Fig. 7a shows PL spectra of ZnFe_2_O_4_ and AZP2 samples. The emission peak observed for both samples observed at 451 nm and indicates to the charge recombination.^45^ As seen, the AZP2 has a weaken PL intensity than the pure ZnFe_2_O_4_, suggesting reduced electron hole pair recombination rate in the AZP2 matrix.^46,47^ It supports the EIS and transient photocurrent findings. The specific surface area of synthesized ZnFe_2_O_4_ and AZP2 nanostructures are measured using N_2_ adsorption–desorption isotherm BET studies and depicted in Fig. 7b. The AZP2 material has a specific surface area of 12.34 m^2^ g^−1^, higher than the 7.41 m^2^ g^−1^ obtained for pure ZnFe_2_O_4_. Booted surface area is an important factor in increasing the photocatalytic activity of nanomaterials. In other words, the boosted surface area in nanomaterials increases the adsorption capacity for the pollutants on the material surface and active photocatalytic sites number, enhancing pollutant decomposition.^48,49^ The photodegradation of MB and RhB dyes in aqueous medium was performed under simulated sunlight illumination for the AZP2 sample as the best photocatalyst was measured and depicted in Fig. 8a and b, respectively. The AZP2 plasmonic nanocomposites degraded 99.6% and 94.7% of MB and RhB molecules after exposure to light illumination for 60 min, respectively. Fig. 8c shows the degradation kinetics (*C*_*t*_/C_0_) for different photocatalysts to decompose MB dye during 60 min illumination. As shown, by increasing amounts of Ag NPs in Ag@ZnFe_2_O_4_–PAni structure, its photocatalyst activity is reduced; indicating optimum amounts of AgNO_3_ solution during synthesis process is 5 mM. As represented in Fig. 8d, the photocatalytic activity of photocatalysts are follows the trend of AZP2 > AZP1 > AZP3 > ZnFe_2_O_4_–PAni > ZnFe_2_O_4_. It indicates that the employed method in here to advance photocatalytic performance of ZnFe_2_O_4_ has been very effective.

**Fig. 9:**
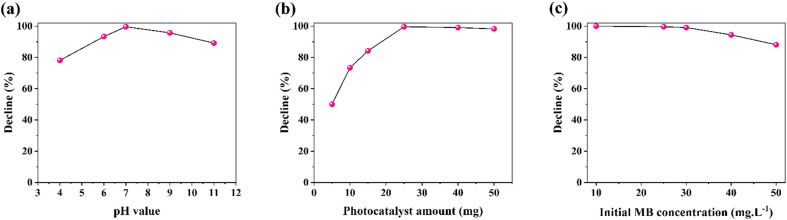
(a) Effect of pH solution, (b) effect of photocatalyst amount and (c) effect of initial MB concentration on the photodegradation performance for AZP2.

The solution pH value plays an important role in the photocatalytic-based reactions. The pH adjusting alters the interaction between the photocatalyst and dye molecules. Here, the photocatalytic activity of AZP2 nanostructures enhances from 78.1% to 99.6% as the pH value increases from 4 to 7 (Fig. 9a). By increasing the pH value to non-acidic conditions, the photocatalytic efficiency reduces to 89.2 (pH 11). Under acidic conditions (pH < 7), both Ag@ZnFe_2_O_4_–PAni and MB are positively charged, which results in a repulsive interaction in the solution and reduces photocatalytic activity. In neutral conditions, appropriate interactions between positively charged photocatalysts and MB enhance the photocatalytic activity. Under alkaline conditions, a slight reduction in the photocatalytic efficiency is observed, which is due to the interactions between anionic MB negatively charged photocatalysts. Next, effect of the initial AZP2 amount on the photocatalytic activity was investigated (Fig. 9b). For this aim, 5, 10, 15, 25, 40, and 50 mg of AZP2 nanostructures were dispersed in 100 mL of 25 ppm MB aqueous solution and their photocatalytic activities over 60 min light illumination were monitored. By enhancing the AZP2 amount, the MB photodegradation efficiency increased. The MB photodegradation efficiency started declining by raising the AZP2 amount to >40 mg, possibly due to the photocatalysts aggregation or more light scattering from photocatalyst nanoparticles.

Furthermore, effect of the initial MB concentration on the AZP2 photocatalytic performance was studied by varying MB concentration from 10 to 50 mg L^−1^ and probing their degradation efficiency. As shown in Fig. 9c, the photodegradation performance reduced from 100% (for 10 mg L^−1^) to 88.1% (50 mg L^−1^). The observed decline in performance is possibly due to the saturation of photocatalytic sites of AZP2 in high amounts of MB.

**Fig. 10:**
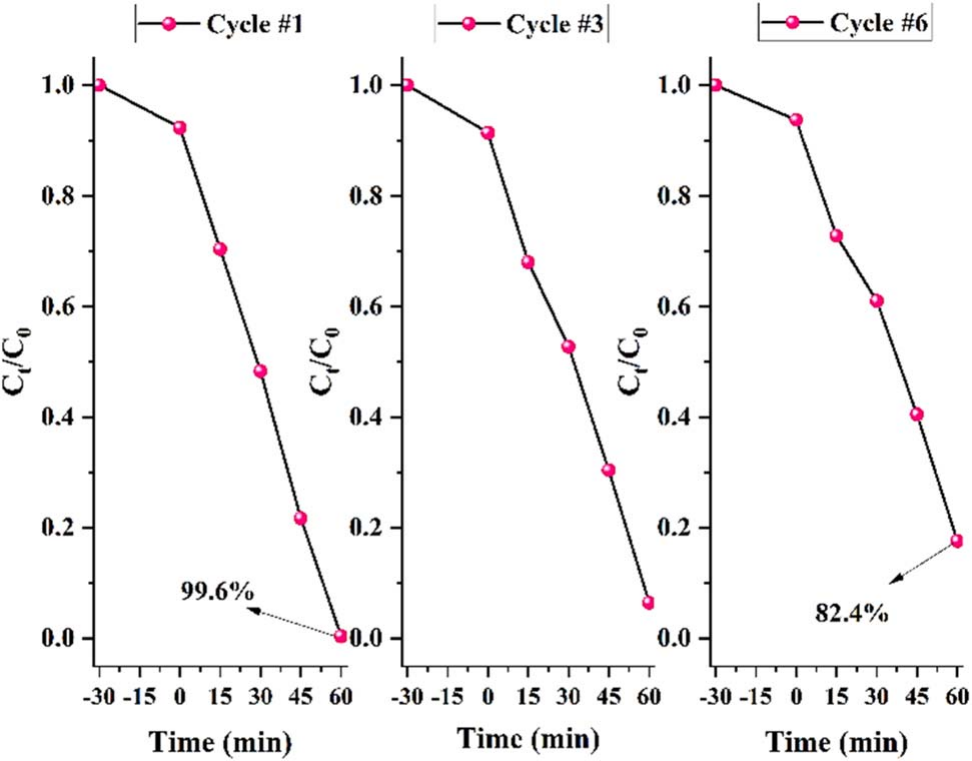
Stability test of photodegradation of MB for AZP2.

Reusability experiments were conducted for six successive runs (Fig. 10). After each photodegradation run, the AZP2-contained solution was centrifuged at 4000 rpm for 6 min. The collected AZP2 was washed with deionized water and methanol, followed by drying for 6 h at 75 °C. The dried AZP2 was again used for reusability test. As can be seen, a photocatalytic performance of 99.6% and 96.6% was recorded for the first and second cycles; however, in total a ∼17% decline in photocatalytic performance was observed after six runs. The reusability test implies that the Ag@ZnFe_2_O_4_–PAni plasmonic nanocomposites can be used for photodegradation of industrial aqueous wastes.

**Fig. 11:**
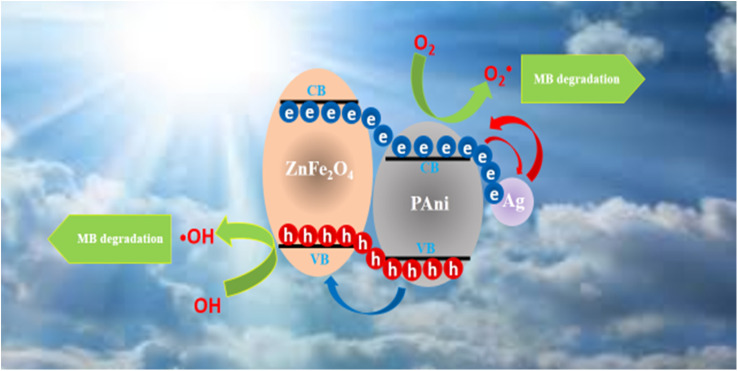
Schematic view for possible photocatalytic mechanism behind Ag@ZnFe_2_O_4_–PAni nanocomposites for photo-degradation of dye molecules.

The possible photodegradation mechanism of the Ag@ZnFe_2_O_4_–PAni system is depicted in Fig. 11. By exposing sunlight illumination to the Ag@ZnFe_2_O_4_–PAni/dye aqueous solution, the ZnFe_2_O_4_ valence band (VB) electrons absorb light energy and generate hot electrons. Next, these hot electrons inject into the conduction band of PAni polymer, then moved to Ag NPs surface. Theses hot electrons react with surrounding O_2_ and form superoxide radicals (O_2_˙). The O_2_˙ radicals assist to form the hydroxyl radicals (˙OH). In addition, the holes from the PAni molecules transfer to the VB of ZnFe_2_O_4_, which contribute to the ˙OH radicals formation. Then, the ˙OH and O_2_˙ radicals decompose the dye molecules.

Table 1 summarizes the kinetic rate values provided for several types of nanocomposites and compares them with the values acquired in this work.

**Table 1:** Compare the obtained kinetic rate for MB degradation with literature

Photocatalyst	Kinetic rate (min^−1^)	Photocatalyst	Kinetic rate (min^−1^)
CeO_2_/GO/PAM^50^	0.0259	ZnO^51^	0.0300
MnTiO_3_/TiO_2_ (ref. 52)	0.0059	Bi_2_O_3_/MoSe_2_ (ref. 53)	0.0455
NiO/Ag/TiO_2_ (ref. 54)	0.0312	rGO/WO_3_ (ref. 55)	0.0073
Ag_2_O^56^	0.0319	g-C_3_N_4_/CoFe_2_O_4_ (ref. 57)	0.0190
CdSe^58^	0.0380	This study	0.0406

## 4 Conclusions

In the current study, ZnFe_2_O_4_ photocatalytic activity was increased by developing Ag@ZnFe_2_O_4_–PAni plasmonic nanostructures. The synthesized Ag@ZnFe_2_O_4_–PAni nanostructures was employed to photodegradation of MB and RhB dyes under simulated sunlight illumination. Results showed the ternary nanostructures exhibit higher photocatalytic efficiency than that of the pure ZnFe_2_O_4_ ferrites. After 60 min light illumination, Ag@ZnFe_2_O_4_–PAni plasmonic nanostructures decomposed 99.6% of MB dye. Incorporating Ag NPs into to ZnFe_2_O_4_–PAni nanocomposites boosted light-harvesting photocatalyst and reduced energy bandgap from 2.01 eV to 1.92 eV. These phenomenons increased the electron–hole production rate in ZnFe_2_O_4_ by exposure it to light. Moreover, contribution Ag NPs and PAni to charge transfer mechanisms boosted charge separation during photocatalytic process. The Ag@ZnFe_2_O_4_–PAni plasmonic nanostructures offered larger surface area and photocatalytic sites than the pure ZnFe_2_O_4_. Overall, enlarged surface area, increased electron–hole production rate, and boosted charge separation are the origins of photocatalytic improvement of ZnFe_2_O_4_. Furthermore, the Ag@ZnFe_2_O_4_–PAni photocatalyst with its considerable reusability can be used in industries to photodegrade their aqueous wastes. In addition, the obtained results show that by modification ZnFe_2_O_4_ materials with metallic dopants and by developing ZnFe_2_O_4_–polymer hybrid systems can design efficient photocatalyst materials to waste water treatments.

## Data availability

The datasets used and/or analysed during the current study available from the corresponding author on reasonable request.

## Author contributions

Conceptualization, methodology, formal analysis, investigation, data curation, validation; visualization, original draft preparation, writing–review and editing M. K. A. M., A. M. N., S. S. J., R. I. F., O. A. N. All authors reviewed the manuscript.

## Conflicts of interest

The authors declare no conflict of interest.
